# Supplementary material for: Observation of emergent momentum–time skyrmions in parity–time-symmetric non-unitary quench dynamics
Source: Nat Commun. 2019 May 23;10:2293. doi: 10.1038/s41467-019-10252-7 (PMC6533298; doi:10.1038/s41467-019-10252-7)
Supplement: Supplementary file 1 — Supplementary Information [file 41467_2019_10252_MOESM1_ESM.pdf]

Supplementary Information for "Observation of emergent momentum-time skyrmions in parity-time-symmetric non-unitary quench dynamics" by Wang et al.

SUPPLEMENTARY FIGURES

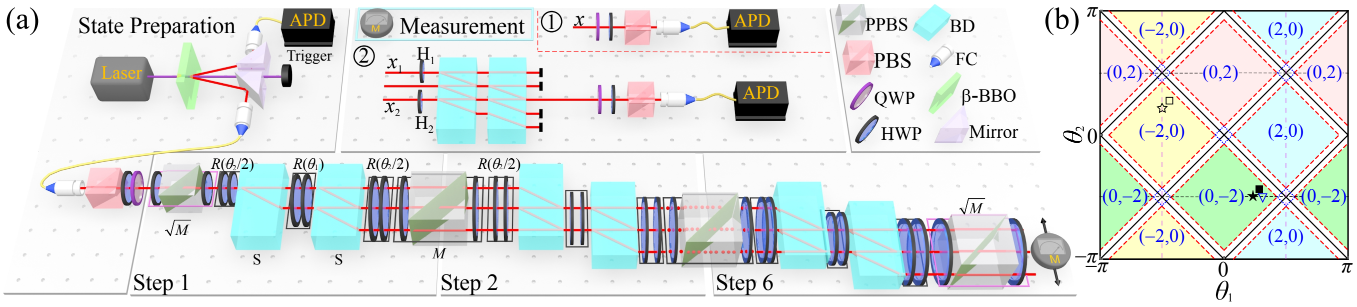

Supplementary Figure 1. Experimental setup for U' and the complete phase diagram. **(a)** Experimental setup for detecting momentum-time skyrmions governed by U'. **(b)** Phase diagram for QWs governed by the Floquet operators (U, U') with the corresponding winding numbers (ν, ν') as functions of coin parameters (θ_1, θ_2). Solid black lines are the topological phase boundary, dashed red lines represent boundaries between $\mathcal{PT}$-symmetry-unbroken and broken regimes. The open black star represents coin parameters of U'^{i}. Blue open triangle and black open square correspond to coin parameters of U'^{f} in Supplementary Figs. 2 and 3, respectively. The black filled star and black filled square respectively correspond to coin parameters of U^{i} and U^{f} in Supplementary Figure 4.

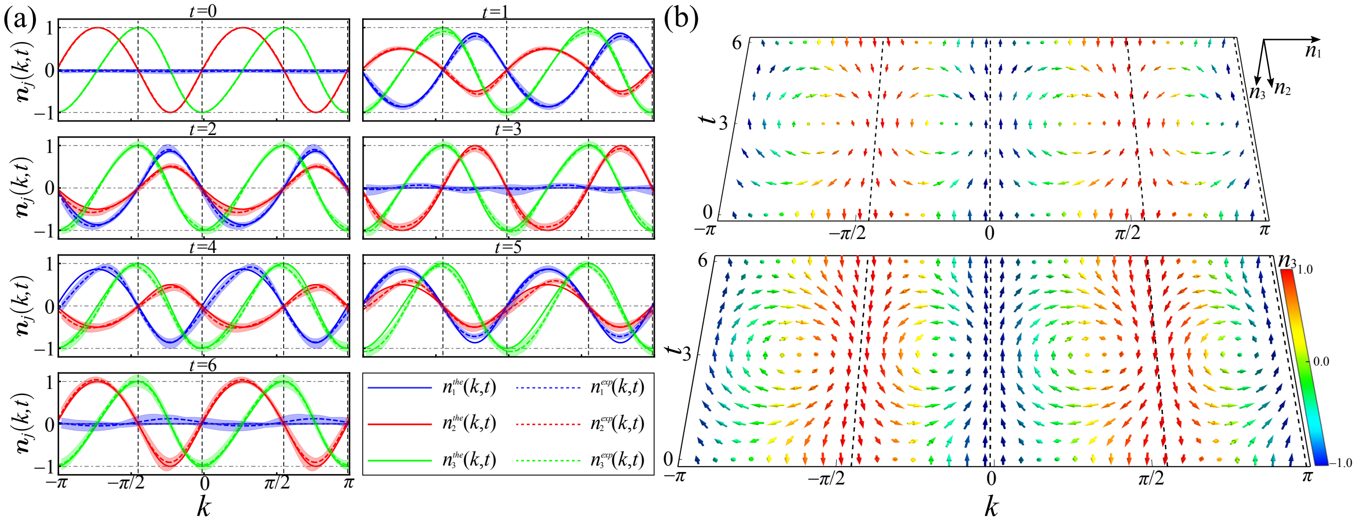

Supplementary Figure 2. Experimental results of $\mathbf{n}(k,t)$ for the $\mathcal{PT}$-symmetric preserving dynamics governed by U'. **(a)** Time-evolution of $\mathbf{n}(k,t)$ for quench processes between an initial non-unitary Floquet operator U' characterized by $(\theta_1^{\mathrm{i}} = -\pi/2, \theta_2^{\mathrm{i}} = \pi/4)$ and a final Floquet operator characterized by $(\theta_1^{\mathrm{f}} = \arcsin(\frac{1}{\alpha}\cos\frac{\pi}{6}), \theta_2^{\mathrm{f}} = -\pi/2)$; The period of oscillations is $t_0 = 6$ for all k. Fixed points are located at $\{-0.4519\pi, -0.0124\pi, 0.5481\pi, 0.9876\pi\}$, which are slightly different from Fig. 3(b) in the main text. **(b)** Experimental (upper) and numerical (lower) results of spin texture $\mathbf{n}(k,t)$ in the momentum-time space. The color code represents the value of $n_3(k,t)$.

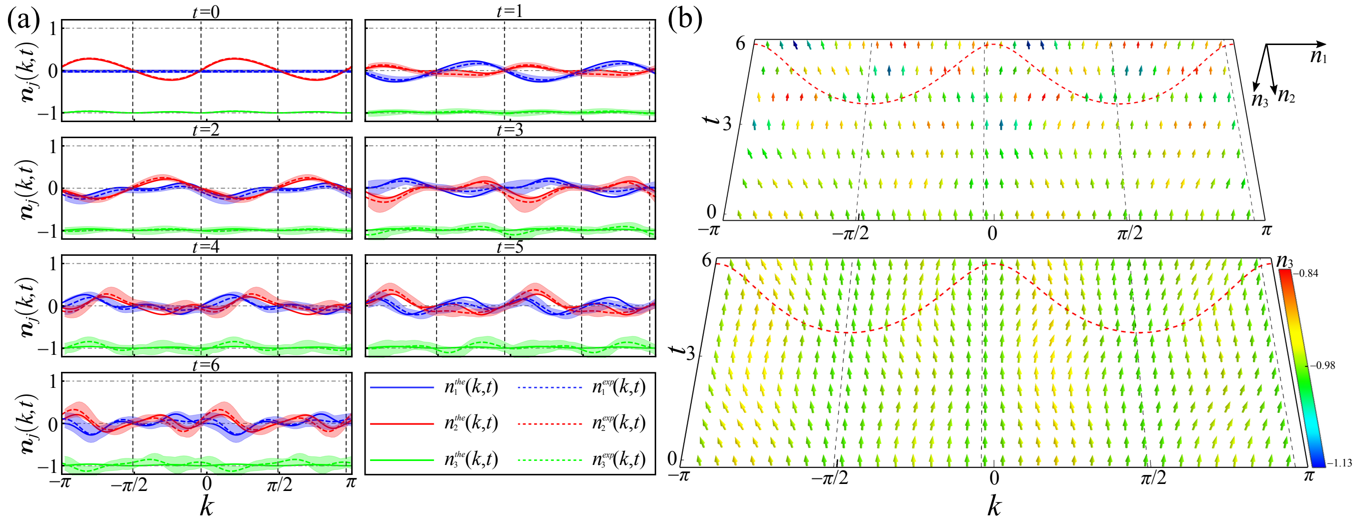

Supplementary Figure 3. Experimental results of $\mathbf{n}(k,t)$ driven by U' between the same initial and final topological phases. **(a)** Time-evolution of $\mathbf{n}(k,t)$ for quench processes between an initial non-unitary Floquet operator U' characterized by ($\theta_1^{\mathrm{i}} = -\pi/2, \theta_2^{\mathrm{i}} = \pi/4$) and a final Floquet operator characterized by ($\theta_1^{\mathrm{f}} = -9\pi/20, \theta_2^{\mathrm{f}} = 7\pi/25$). Fixed points are located at $\{-0.5101\pi, -0.0399\pi, 0.4899\pi, 0.9601\pi\}$. **(b)** Experimental (upper) and numerical (lower) results of spin texture $\mathbf{n}(k,t)$ in the momentum-time space. The color code represents the value of $n_3(k,t)$.

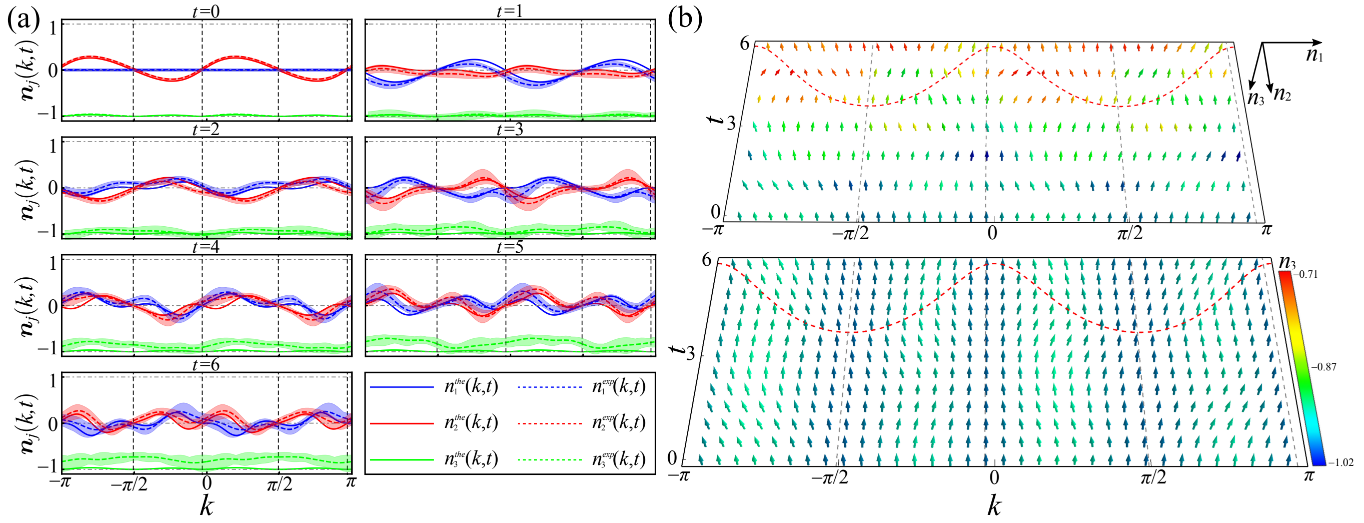

Supplementary Figure 4. Experimental results for the $\mathcal{PT}$-symmetry-unbroken QW dynamics governed by U between the same topological phase. **(a)** Time-evolution and **(b)** spin textures of $\mathbf{n}(k,t)$ in the momentum-time space for a quench process between the initial non-unitary Floquet operator given by ($\theta_1^{\mathrm{i}} = \pi/4, \theta_2^{\mathrm{i}} = -\pi/2$) and the final Floquet operator given by ($\theta_1^{\mathrm{f}} = 7\pi/25, \theta_2^{\mathrm{f}} = -9\pi/20$). Fixed points are located at $\{-1.0319\pi, -0.5069\pi, -0.0319\pi, 0.4931\pi\}$. Red dashed lines in (b) show the momentum-dependent period π/E_k^{f} for the oscillations of $\mathbf{n}(k,t)$, which, together with fixed points in momentum space, mark the boundary of the momentum-time submanifolds. The color code represents the value of $n_3(k,t)$.

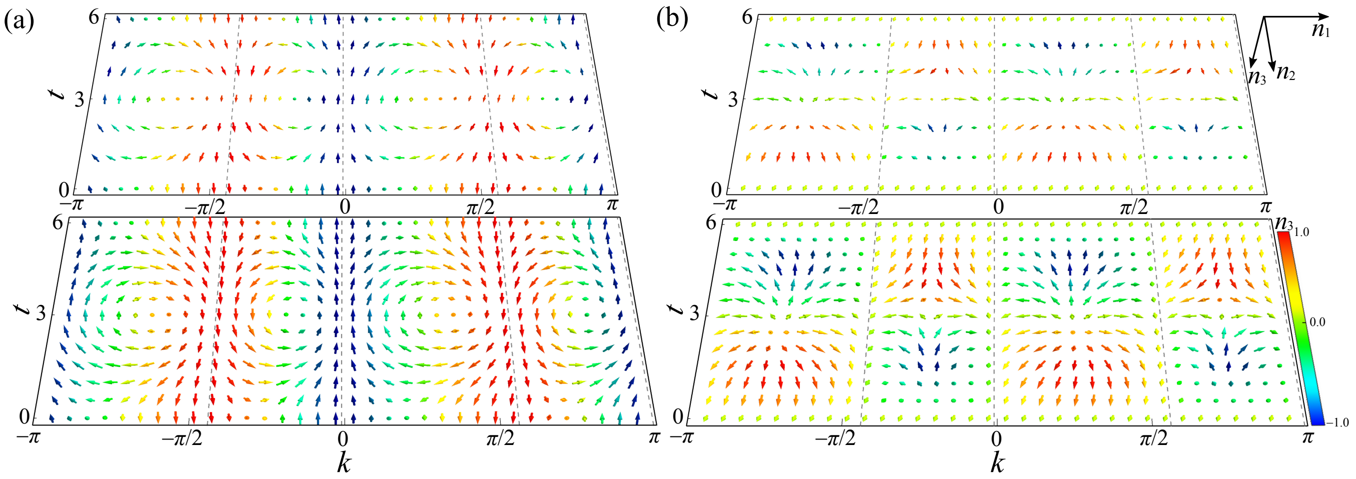

Supplementary Figure 5. Experimental results of spin textures $\mathbf{n}(k,t)$ **(a)** and normalized $\mathbf{n}'(k,t)$ **(b)**. The quench process is the same as that in Fig. 4(b) of the main text. Locations of fixed points in **(a)** are the same as those in **(b)**. The color codes represent values of $n_3(k,t)$ and $n'_3(k,t)$, respectively.

SUPPLEMENTARY NOTES

Supplementary Note 1: Experimental detection of the matrix element $\langle\psi_{x_2}(t)|\,\sigma_i\,|\psi_{x_1}(t)\rangle$

In this section, we detail the experimental detection of matrix elements $\langle\psi_{x_2}(t)|\,\sigma_i\,|\psi_{x_1}(t)\rangle$ $(i=0,1,2,3)$, which are critical for the detection of momentum-time skyrmions.

For matrix elements with $x_1 = x_2 = x$, we perform polarization analysis on each lattice site in the basis $\{|H\rangle, |V\rangle, |L\rangle = (|H\rangle - i\,|V\rangle)/\sqrt{2}, |D\rangle = (|H\rangle + |V\rangle)/\sqrt{2}\}$. Denoting probabilities of photons measured in the four basis states respectively as $P_{\mathrm{H}}(x,t)$, $P_{\mathrm{V}}(x,t)$, $P_{\mathrm{L}}(x,t)$ and $P_{\mathrm{D}}(x,t)$, we have the matrix elements

$$\begin{aligned}
\langle\psi_x(t)|\,\sigma_0\,|\psi_x(t)\rangle &= P_{\mathrm{H}}(x,t) + P_{\mathrm{V}}(x,t), \\
\langle\psi_x(t)|\,\sigma_1\,|\psi_x(t)\rangle &= 2P_{\mathrm{D}}(x,t) - P_{\mathrm{H}}(x,t) - P_{\mathrm{V}}(x,t), \\
\langle\psi_x(t)|\,\sigma_2\,|\psi_x(t)\rangle &= -2P_{\mathrm{L}}(x,t) + P_{\mathrm{H}}(x,t) + P_{\mathrm{V}}(x,t), \\
\langle\psi_x(t)|\,\sigma_3\,|\psi_x(t)\rangle &= P_{\mathrm{H}}(x,t) - P_{\mathrm{V}}(x,t).
\end{aligned} \tag{1}$$

For matrix elements with $x_1 \neq x_2$, we need to measure

$$\begin{aligned}
\langle\psi_{x_2}(t)|\,\sigma_0\,|\psi_{x_1}(t)\rangle &= a^*_{x_2}(t)a_{x_1}(t) + b^*_{x_2}(t)b_{x_1}(t), \\
\langle\psi_{x_2}(t)|\,\sigma_1\,|\psi_{x_1}(t)\rangle &= a^*_{x_2}(t)b_{x_1}(t) + b^*_{x_2}(t)a_{x_1}(t), \\
\langle\psi_{x_2}(t)|\,\sigma_2\,|\psi_{x_1}(t)\rangle &= -ia^*_{x_2}(t)b_{x_1}(t) + ib^*_{x_2}(t)a_{x_1}(t), \\
\langle\psi_{x_2}(t)|\,\sigma_3\,|\psi_{x_1}(t)\rangle &= a^*_{x_2}(t)a_{x_1}(t) - b^*_{x_2}(t)b_{x_1}(t),
\end{aligned} \tag{2}$$

where we have denoted $|\psi_x(t)\rangle = \left[a_x(t), b_x(t)\right]^{\mathrm{T}}$. Here instead of projective measurements, we perform interference-based measurements. As illustrated in Fig. 1 of the main text, photons in spatial modes x_1 and x_2 are injected into the same spatial mode by passing through half-wave plates (HWPs, H_1 and H_2) and beam displacers (BDs). After passing through H_1 and H_2 with specific setting angles, the polarization states of the photons are prepared into one of the following four states:

$$\begin{aligned}
|\phi_1\rangle_{\mathrm{c}} &\propto \Big[a_{x_1}(t), a_{x_2}(t)\Big]^{\mathrm{T}} \text{ when } \mathrm{H}_1 \text{ is at } 0, \mathrm{H}_2 \text{ is at } 45^\circ, \\
|\phi_2\rangle_{\mathrm{c}} &\propto \Big[b_{x_1}(t), -b_{x_2}(t)\Big]^{\mathrm{T}} \text{ when } \mathrm{H}_1 \text{ is at } 45^\circ, \mathrm{H}_2 \text{ is at } 0 \\
|\phi_3\rangle_{\mathrm{c}} &\propto \Big[b_{x_1}(t), a_{x_2}(t)\Big]^{\mathrm{T}} \text{ when } \mathrm{H}_1 \text{ is at } 45^\circ, \mathrm{H}_2 \text{ is at } 45^\circ, \\
|\phi_4\rangle_{\mathrm{c}} &\propto \Big[a_{x_1}(t), b_{x_2}(t)\Big]^{\mathrm{T}} \text{ when removing } \mathrm{H}_1 \text{ and } \mathrm{H}_2.
\end{aligned} \tag{3}$$

We then apply a projective measurement $\{|L\rangle\langle L|, |D\rangle\langle D|\}$ with a quarter-wave plate (QWP), a HWP and a polarizing beam splitter (PBS) to obtain probabilities of photons in the basis states $\{|L\rangle, |D\rangle\}$. Depending on their polarization states $|\phi_j\rangle_{\mathrm{c}}$ $(j=1,2,3,4)$ prior to the projective measurement, we denote the measured probabilities as $P^j_L(x_1,x_2,t)$ and $P^j_D(x_1,x_2,t)$, respectively.

We are then able to calculate both the real and imaginary parts of $\langle\psi_{x_2}(t)|\,\sigma_i\,|\psi_{x_1}(t)\rangle$ $(i=0,1,2,3)$ through

$$\begin{aligned}
\mathrm{Re}\big[\langle\psi_{x_2}(t)|\,\sigma_0\,|\psi_{x_1}(t)\rangle\big] &= P^1_{\mathrm{D}}(x_1,x_2,t) - P^2_{\mathrm{D}}(x_1,x_2,t) - \frac{P_{\mathrm{H}}(x_1,t) + P_{\mathrm{H}}(x_2,t) - P_{\mathrm{V}}(x_1,t) - P_{\mathrm{V}}(x_2,t)}{2}, \\
\mathrm{Im}\big[\langle\psi_{x_2}(t)|\,\sigma_0\,|\psi_{x_1}(t)\rangle\big] &= P^1_{\mathrm{L}}(x_1,x_2,t) - P^2_{\mathrm{L}}(x_1,x_2,t) - \frac{P_{\mathrm{H}}(x_1,t) + P_{\mathrm{H}}(x_2,t) - P_{\mathrm{V}}(x_1,t) - P_{\mathrm{V}}(x_2,t)}{2}, \\
\mathrm{Re}\big[\langle\psi_{x_2}(t)|\,\sigma_1\,|\psi_{x_1}(t)\rangle\big] &= P^3_{\mathrm{D}}(x_1,x_2,t) + P^4_{\mathrm{D}}(x_1,x_2,t) - \frac{P_{\mathrm{V}}(x_1,t) + P_{\mathrm{H}}(x_2,t) + P_{\mathrm{H}}(x_1,t) + P_{\mathrm{V}}(x_2,t)}{2}, \\
\mathrm{Im}\big[\langle\psi_{x_2}(t)|\,\sigma_1\,|\psi_{x_1}(t)\rangle\big] &= P^3_{\mathrm{L}}(x_1,x_2,t) + P^4_{\mathrm{L}}(x_1,x_2,t) - \frac{P_{\mathrm{V}}(x_1,t) + P_{\mathrm{H}}(x_2,t) + P_{\mathrm{H}}(x_1,t) + P_{\mathrm{V}}(x_2,t)}{2}, \\
\mathrm{Re}\big[\langle\psi_{x_2}(t)|\,\sigma_2\,|\psi_{x_1}(t)\rangle\big] &= P^3_{\mathrm{L}}(x_1,x_2,t) - P^4_{\mathrm{L}}(x_1,x_2,t) - \frac{P_{\mathrm{V}}(x_1,t) + P_{\mathrm{H}}(x_2,t) - P_{\mathrm{H}}(x_1,t) - P_{\mathrm{V}}(x_2,t)}{2}, \\
\mathrm{Im}\big[\langle\psi_{x_2}(t)|\,\sigma_2\,|\psi_{x_1}(t)\rangle\big] &= P^4_{\mathrm{D}}(x_1,x_2,t) - P^3_{\mathrm{D}}(x_1,x_2,t) + \frac{P_{\mathrm{V}}(x_1,t) + P_{\mathrm{H}}(x_2,t) - P_{\mathrm{H}}(x_1,t) - P_{\mathrm{V}}(x_2,t)}{2}, \\
\mathrm{Re}\big[\langle\psi_{x_2}(t)|\,\sigma_3\,|\psi_{x_1}(t)\rangle\big] &= P^1_{\mathrm{D}}(x_1,x_2,t) + P^2_{\mathrm{D}}(x_1,x_2,t) - \frac{P_{\mathrm{H}}(x_1,t) + P_{\mathrm{H}}(x_2,t) + P_{\mathrm{V}}(x_1,t) + P_{\mathrm{V}}(x_2,t)}{2}, \\
\mathrm{Im}\big[\langle\psi_{x_2}(t)|\,\sigma_3\,|\psi_{x_1}(t)\rangle\big] &= P^1_{\mathrm{L}}(x_1,x_2,t) + P^2_{\mathrm{L}}(x_1,x_2,t) - \frac{P_{\mathrm{H}}(x_1,t) + P_{\mathrm{H}}(x_2,t) + P_{\mathrm{V}}(x_1,t) + P_{\mathrm{V}}(x_2,t)}{2},
\end{aligned} \tag{4}$$

from which we construct the corresponding matrix elements.

Supplementary Note 2: Quench dynamics in the alternative time frame

As we have discussed in Methods, a complete description of topological properties of the system requires another winding number associated with the Floquet operator U' in a different time frame, with

$$U' = M^{\frac{1}{2}} R\left(\frac{\theta_2}{2}\right) SR(\theta_1) SR\left(\frac{\theta_2}{2}\right) M^{\frac{1}{2}}, \tag{5}$$

where $M^{\frac{1}{2}} = \mathbb{1}_{\mathrm{w}} \otimes \left(|+\rangle\langle+| + (1-p)^{\frac{1}{4}} |-\rangle\langle-|\right)$. It is straightforward to show that U' has passive parity-time ($\mathcal{PT}$) symmetry, similar to U in the main text. Similar to the definition of the winding number ν in Methods, we define the winding number associated with U' as ν'. Following the arguments and discussions in Ref. [1], it is easy to show that $(\nu_0, \nu_\pi) = [(\nu + \nu')/2, (\nu - \nu')/2]$, where topological invariants ν_0 and ν_π are related to the number of edge states with quasienergies $\mathrm{Re}\,\epsilon = 0$ and $\mathrm{Re}\,\epsilon = \pi$, respectively, through the bulk-boundary correspondence. We have numerically checked that edge states in inhomogeneous quantum walks are consistent with the topological invariants (ν_0, ν_π). However, as we explicitly demonstrate below, for quench dynamics of homogeneous quantum walks (QWs), it is sufficient to choose a single time frame and focus on the corresponding bulk winding number.

To illustrate quench dynamics in the alternative time frame, we first plot the phase diagram in Supplementary Figure 1, where labels correspond to (ν, ν'). We then experimentally implement U' and simulate quench dynamics starting from a localized initial eigenstate of U'^{i} (open black star in Supplementary Figure 1) with $\nu'^{\mathrm{i}} = 0$ and evolving it under U'^{f} with different parameters. As illustrated in Supplementary Figs. 2 and 3, skyrmion structure emerges only when the winding number ν'^{f} of U'^{f} differs from ν'^{i}. We have therefore experimentally confirmed that choosing different time frames does not qualitatively change the relation between the pre- and post-quench topological invariants and the emergence of skyrmion structures. Note that, similar to the case of U, skyrmion structures would also disappear when the system is quenched into $\mathcal{PT}$-symmetry-broken regimes. Therefore, all our previous conclusions on fixed points, dynamic skyrmions, and $\mathcal{PT}$ symmetry are qualitatively the same for both time frames, whereas spin textures and locations of fixed points are quantitatively different.

Supplementary Note 3: Quench between FTPs with the same winding number

As we have discussed in the main text, when the system is quenched between FTPs with the same winding number, skyrmion-lattice structures are no longer present. This is shown in Supplementary Figure 4, where we set the coin parameters for U^{f} as $(\theta_1^{\mathrm{f}} = 7\pi/25, \theta_2^{\mathrm{f}} = -9\pi/20)$. The initial state and the loss parameter p are the same as those in Fig. 3(b) of the main text. Therefore, the post-quench FTP is in the $\mathcal{PT}$-symmetry unbroken regime with $\nu^{\mathrm{f}} = 0$ and belongs to the same phase regime for U^{i}. As shown in Supplementary Figure 4(a), dynamics of $\mathbf{n}(k,t)$ is still oscillatory; however, skyrmion-lattice structures are no longer present in Supplementary Figure 4(b). Note that, as $\theta_{1,2}^{\mathrm{f}}$ are not chosen along the purple dashed lines in Fig. 2(a), the corresponding quasienergy band E_k^{f} is not flat, which leads to oscillations of $\mathbf{n}(k,t)$ with momentum-dependent periods. This is shown in Supplementary Figure 4(b).

Supplementary Note 4: Choice of density matrix

As we have discussed in the main text and in Methods, the definition of the density matrix $\rho(k,t)$ in Equation (5) of the main text under the biorthogonal basis makes the dynamics transparent on the Bloch sphere. Such a definition offers a simple and elegant proof on the relation between topologically protected fixed points and winding numbers of the Floquet operators. An alternative choice of density matrix is $\rho'(k,t) = |\psi_k(t)\rangle\langle\psi_k(t)|$, which is Hermitian and therefore easier to probe experimentally. In fact, we experimentally probe $\rho'(k,t)$ and construct $\rho(k,t)$ through Equation (9) of the main text. In the following, we show that dynamic skyrmion structures should also emerge in $\mathbf{n}'(k,t) = \mathrm{Tr}\left[\rho'(k,t)\boldsymbol{\sigma}\right]$; albeit $\mathbf{n}(k,t)$ generically differs from $\mathbf{n}'(k,t)$. Here $\boldsymbol{\sigma} = (\sigma_1, \sigma_2, \sigma_3)$.

Applying the definition of $\boldsymbol{\tau}$ and making use of the properties of biorthogonal base, it is straightforward to show

that

$$\mathbf{n}(k,t) = \mathrm{Tr}\left[\rho(k,t)\boldsymbol{\tau}\right] = \sum_{\mu,\nu=\pm} c_\mu c_\nu^* e^{-i\epsilon^{\mathrm{f}}_{k,\mu}t + i\epsilon^{\mathrm{f}*}_{k,\nu}t} \langle\phi_\nu|\boldsymbol{\sigma}|\phi_\mu\rangle, \tag{6}$$

$$\mathbf{n}'(k,t) = \mathrm{Tr}\left[\rho'(k,t)\boldsymbol{\sigma}\right] = \sum_{\mu,\nu=\pm} c_\mu c_\nu^* e^{-i\epsilon^{\mathrm{f}}_{k,\mu}t + i\epsilon^{\mathrm{f}*}_{k,\nu}t} \langle\psi^{\mathrm{f}}_{k,\nu}|\boldsymbol{\sigma}|\psi^{\mathrm{f}}_{k,\mu}\rangle, \tag{7}$$

where $\{|\phi_\pm\rangle\} = \{|H\rangle, |V\rangle\}$. Physically, $\mathbf{n}(k,t)$ and $\mathbf{n}'(k,t)$ are related by a basis transformation of the Pauli operators in the summand. As $\left\{|\psi^{\mathrm{f}}_{k,\pm}\rangle\right\}$ are not orthonormal, such a transformation is non-unitary. As a result, $\mathbf{n}'(k,t)$ is not normalized, and one needs to enforce a normalization on $\mathbf{n}'(k,t)$ to visualize it on the Bloch sphere. Even then, characterizing the dynamics of $\mathbf{n}'(k,t)$ on the Bloch sphere remains cumbersome, and it is difficult to establish a connection between dynamic skyrmion structures and winding numbers. Nevertheless, information of topological fixed points and dynamic skyrmion structures are encoded in the coefficients $c_\mu c_\nu^* e^{-i\epsilon^{\mathrm{f}}_{k,\mu}t + i\epsilon^{\mathrm{f}*}_{k,\nu}t}$. Therefore, dynamic topological structures appearing in $\mathbf{n}(k,t)$ should also emerge in $\mathbf{n}'(k,t)$ under the same conditions. We show in Supplementary Figure 5 an explicit comparison of momentum-time skyrmions in $\mathbf{n}(k,t)$ and a normalized $\mathbf{n}'(k,t)$ following the same quench in Fig. 4(b) of the main text. Whereas fixed points and skyrmion structures exist in both cases, it is apparent that the spin textures are quite different.

Finally, we emphasize that it is also possible to directly construct $\rho(k,t)$ from experimental data via non-unitary time evolution of both $|\psi(t)\rangle$ and $|\chi(t)\rangle$ and taking the adjoint of $|\chi(t)\rangle$. Evidently, this is at the cost of additional experiments.

SUPPLEMENTARY REFERENCES

[1] Asbóth, J. K. & Obuse, H. Bulk-boundary correspondence for chiral symmetric quantum walks. *Phys. Rev. B* **88**, 121406(R) (2013).
